# Supplementary figures and images for: Structural and Functional MRI Data Differentially Predict Chronological Age and Behavioral Memory Performance
Source: eNeuro. 2022 Nov 3;9(6):ENEURO.0212-22.2022. doi: 10.1523/ENEURO.0212-22.2022 (PMC9665883; doi:10.1523/ENEURO.0212-22.2022)

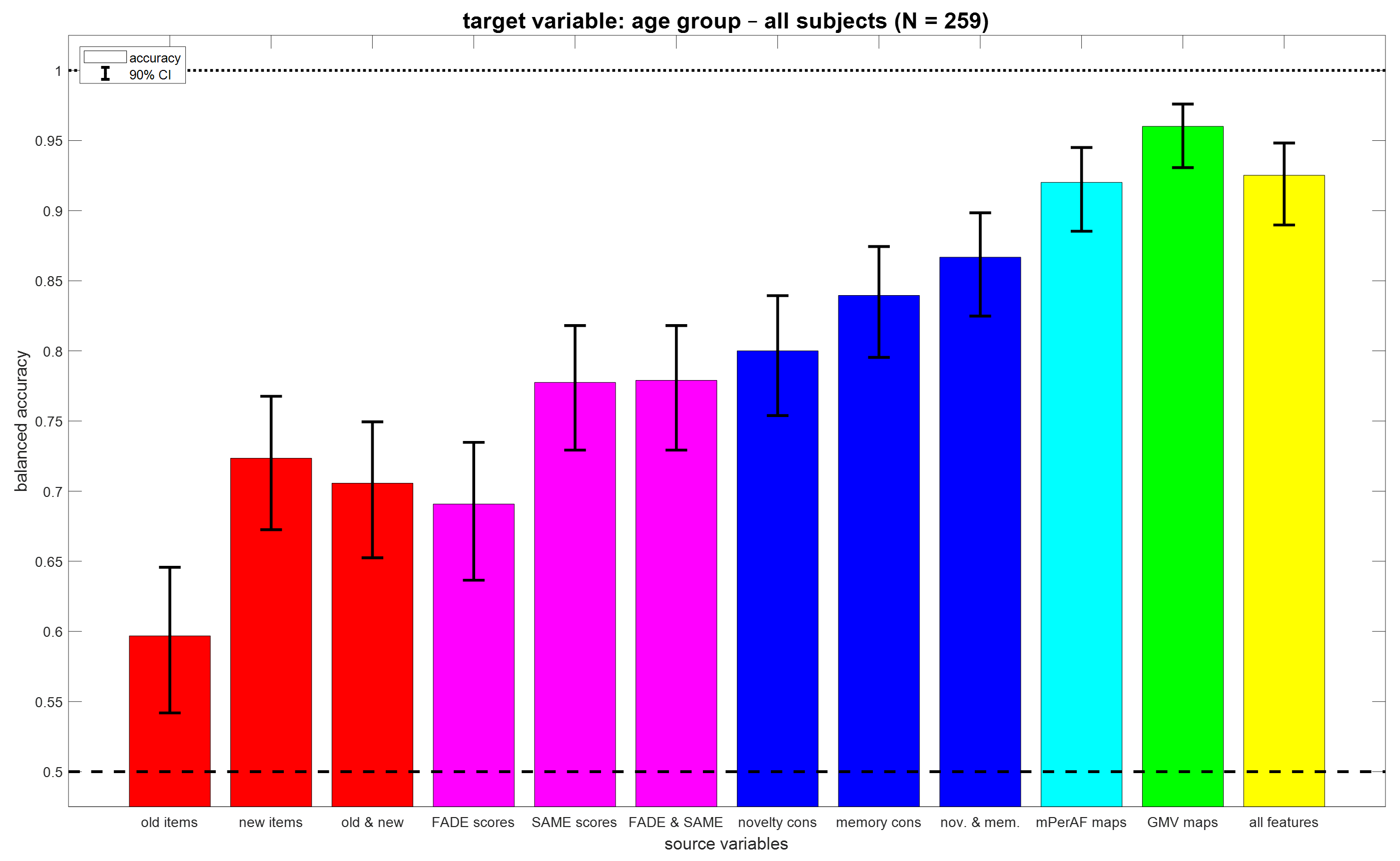

Supplement: Extended Data Figure 2-1 — Classification of age group from different feature sets. Bar plots show accuracy for decoding age group (young vs older) from behavioral data (red), fMRI scores (magenta), task-based fMRI contrasts (blue), resting-state fMRI maps and structural MRI (green), or all features (yellow). Error bars denote 90% CIs; chance level and ceiling performance are indicated by dashed and dotted lines, respectively; x-axis labels are explained in Table 4. Download Figure 2-1, TIF file [file enu-eN-NWR-0212-22-s02.tif]

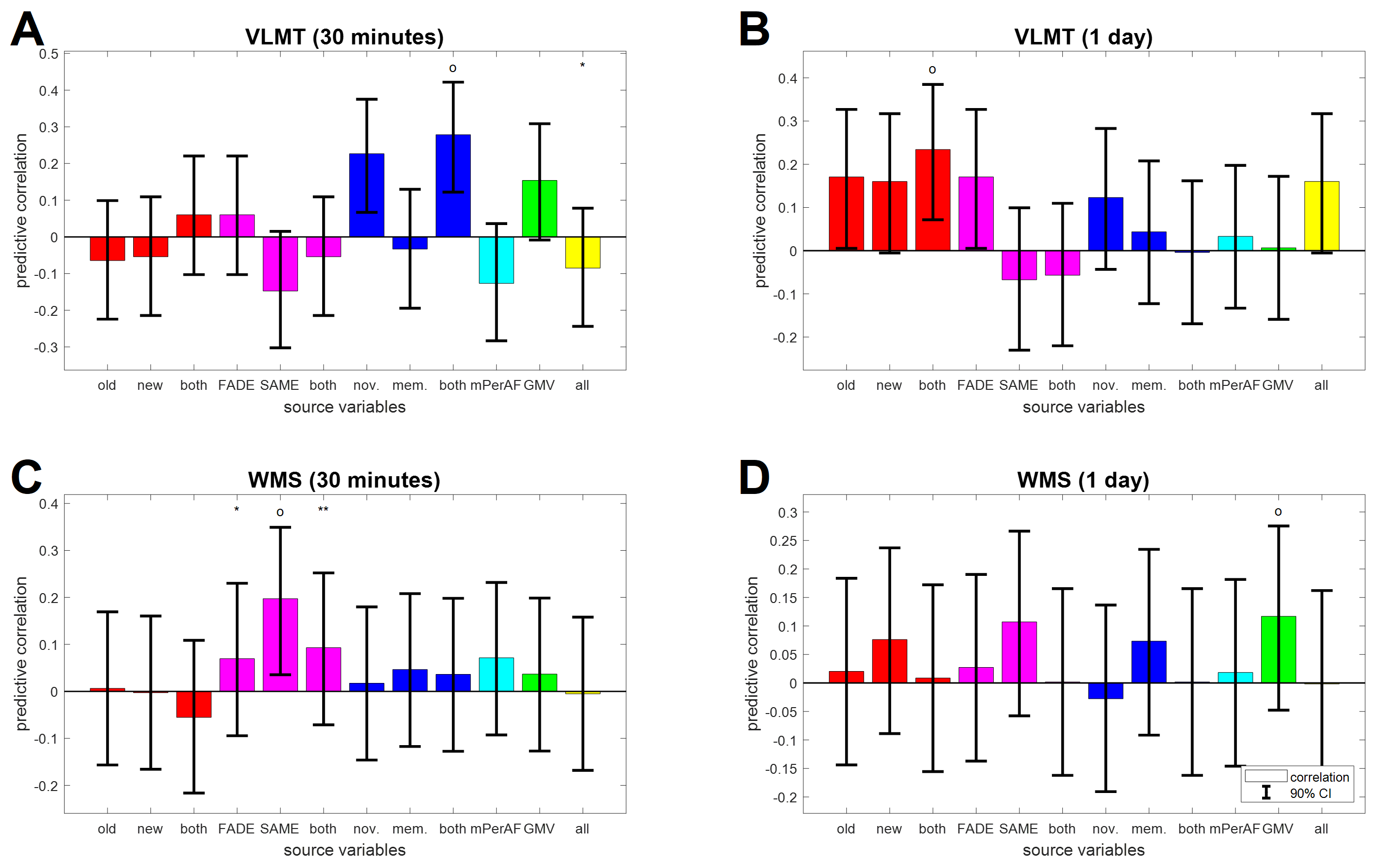

Supplement: Extended Data Figure 4-1 — Reconstruction of independent memory performance in young subjects. Bar plots show correlation coefficients for predicting, in young subjects only, independent measures of memory performance, namely, (A) VLMT items after 30 min, (B) VLMT items after 1 d, (C) WMS points after 30 min, and (D) WMS points after 1 d, from behavioral data (red), fMRI scores (magenta), task-based fMRI contrasts (blue), resting-state fMRI maps (cyan) and structural MRI (green), or all features (yellow). This figure mirrors Figure 4 and the layout follows that of Figure 3C from the main paper. Download Figure 4-1, TIF file. [file enu-eN-NWR-0212-22-s03.tif]

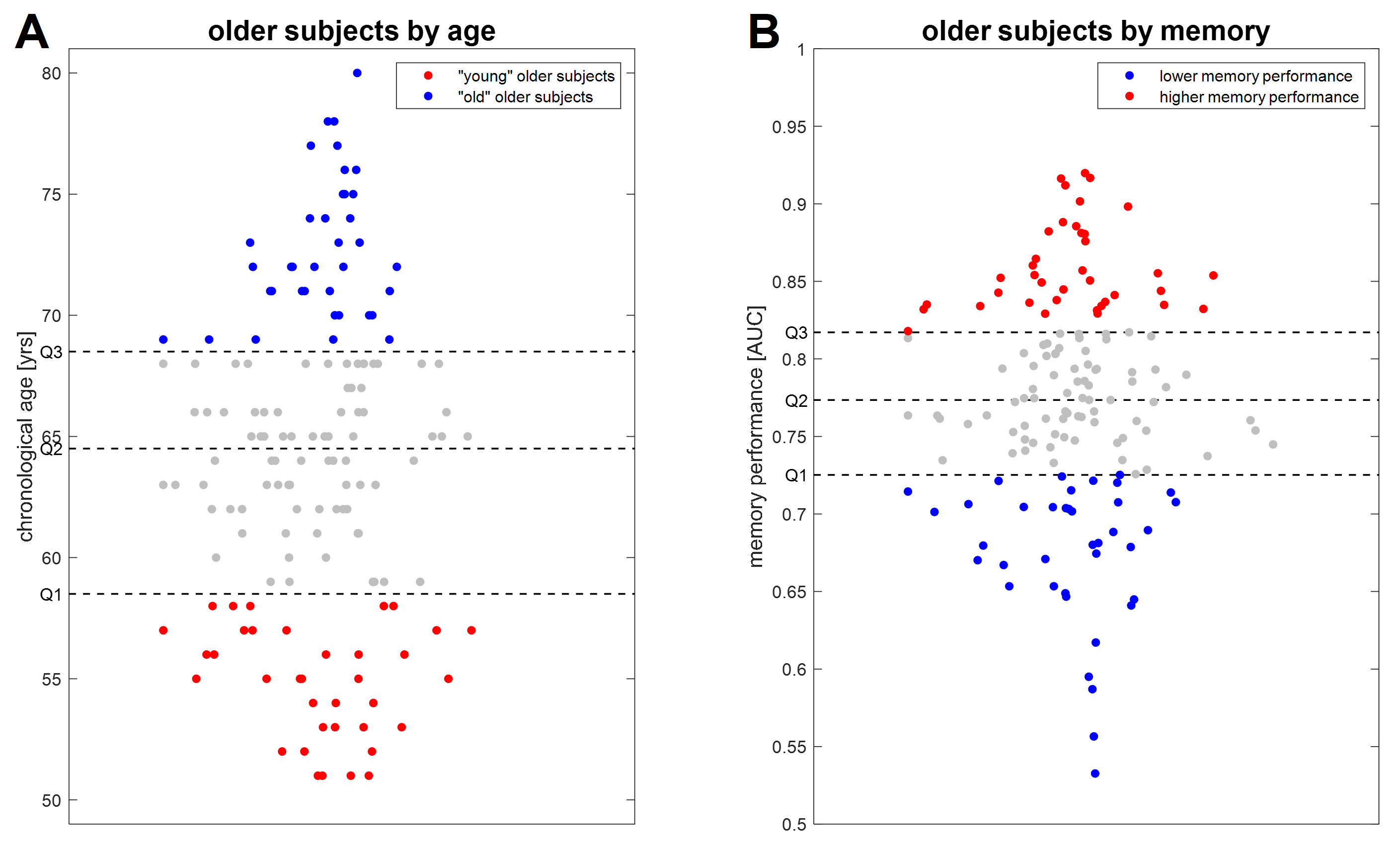

Supplement: Extended Data Figure 5-1 — Separation of older subjects by chronological age and memory performance. Older subjects were partitioned into four groups based on quartiles (dashed black lines) obtained from the observed distributions of (A) chronological age and (B) memory performance. Subjects at the lower and the upper end (red and blue) were used for post hoc analyses reported in Results, Effects of age and memory are specific to structural MRI versus fMRI and Single-value fMRI scores have moderate predictive utility. Download Figure 5-1, TIF file. [file enu-eN-NWR-0212-22-s04.tif]
